# Combination of Conservative and Interventional Therapy Strategies for Intra- and Extrahepatic Cholangiocellular Carcinoma: A Retrospective Survival Analysis

**DOI:** 10.1155/2012/190708

**Published:** 2011-06-08

**Authors:** M. Knüppel, S. Kubicka, A. Vogel, N. P. Malek, M. Schneider, F. Papendorf, T. Greten, J. Wedemeyer, A. Schneider

**Affiliations:** ^1^Departement of Anesthesiology and Intensive Care Medicine, Hannover Medical School, 30625 Hannover, Germany; ^2^Department of Gastroenterology, Hepatology and Endocrinology, Hannover Medical School, 30625 Hannover, Germany; ^3^Centre for Biometry, Medical Informatics and Medical Technology, Institute for Biometry, Hannover Medical School, 30625 Hannover, Germany; ^4^Tumour Centre Hannover, Hannover Medical School, 30625 Hannover, Germany; ^5^Gastrointestinal Malignancies, National Cancer Institute, Bethesda, MD 20892-8322, USA

## Abstract

*Background*. Due to the predominantly advanced stage at the time of diagnosis treatment of cholangiocarcinoma is difficult. Apart from surgical resection, interventional treatment strategies are increasingly used in advanced stage tumours. The aim of the study was a retrospective comparison of the effect of the various forms of treatment on morbidity and mortality. *Method*. A total of 195 patients, received either chemotherapy or a combination of photodynamic therapy (PDT) or transarterial chemoembolization (TACE) and chemotherapy. *Results*. The median survival rate for all patients was 15.6 months, 50.8% were still alive 1 year after diagnosis. Patients, who had previously undergone surgery, survived 17.1 months longer than those without surgical treatment (*P* < .01). Chemotherapy prolonged the survival by 9.2 months (*P* = .47). Palliative patients under combination of chemotherapy and PDT survived on average 1.8 months longer (*P* = .28), with chemotherapy and TACE 9.8 months longer (*P* = .04) compared to chemotherapy alone. *Conclusions*. It appears that surgical treatment and chemotherapy combined with PDT or TACE may prolong survival.

## 1. Introduction

Carcinomas of the bile tract, a malignant neoplasia spreading from the bile duct epithelia, were described for the first time as tumours of the common hepatic duct by Durand-Fardel in 1840. 

20–25% of all tumours are located intrahepatic, 50–60% perihilar, and 20–25% extrahepatic [1, 2]. The classification by Bismuth et al. [3] groups perihilar tumours located in the main branches of the biliary tree into four different categories. Type I tumours are limited to the common bile duct and are located more than 2 cm away from the confluence of right and left hepatic ducts while type II tumours involve the confluence. Type III tumours involve either the right (IIIa) or left (IIIb) hepatic duct while type IV tumours extend to both ducts or are located multifocally. “Klatskin” tumours are those with involvement of the hepatic duct bifurcation [4]. Today, cholangiocarcinoma is the second most frequent primary tumour disease of the liver, nevertheless, the prevalence rate is relatively low (2-3/100,000) compared with other tumours of the gastrointestinal tract [5, 6].

Due to the predominantly advanced tumour stage at the time of diagnosis, therapy of cholangiocarcinoma and gallbladder carcinoma remains difficult [7]. Currently, the only curative treatment is R0 resection [8]. However, in the majority of cases advanced tumour spread and lymph node involvement require a palliative approach. Surgical therapy of perihilar Klatskin tumours depends on the Bismuth classification. Tumour of types I and II are treated with en-bloc resection of the extrahepatic bile ducts and the gallbladder, regional lymphadenectomy, and Roux-Y hepatico-jejunostomy. In case of type III tumours an additional left or right hemihepatectomy is performed, whereas type IV tumours necessitate additional extended hemihepatectomy. The survival rate of patients with intrahepatic tumours is 18–24 months depending on the hilar infiltration [9].

Unresectable bile duct and gallbladder tumours are associated with a very poor prognosis. Apart from the recommendation for best supportive care, adequate drainage of the bile ducts with plastic or metal stent via endoscopic retrograde cholangiography (ERC) is an important element of the mainly palliative therapy [8, 10].

Bile duct tumours as well as gallbladder tumours are moderately chemotherapy-sensitive tumours. So far there is no standard protocol, thus patients in good general condition or with tumour-associated symptoms should be included in clinical trials evaluating palliative chemotherapy. For example, prolonged survival (4 months) and higher quality of life are reported for a small prospective randomised trial evaluating the combination of 5-FU/Leucovorin and Etoposide versus “best supportive care”; however these data are without significance [11]. Other study protocols cover treatment with gemcitabine or 5-FU as monotherapy as well as in combination with cisplatin and the combination of capecitabine with cisplatin/5-FU. The available data world-wide show that there might be a possibility to document the benefit of certain substances in trials with sufficiently large numbers of patients. Apart from the application of chemotherapeutic drugs interventional procedures applied and evaluated, for example transarterial chemoembolisation (TACE) [12, 13].

Another procedure is photodynamic therapy (PDT), whereby patients are given a photosensitizing agent (for example photofrin), which increasingly accumulates in malignant cells. Thereafter, transpapillary or percutaneous radiation with light of a specific wave length activates the sensitizing agent and generates reactive oxygen radicals. This leads to destruction of the tumour cells. In the early 1990's McCaughan reported on the first successful application of photodynamic therapy in the treatment of bile duct carcinoma [14]. A prospective study by Ortner et al. in the late 1990's showed a significant positive effect of PDT with Photofrin in combination with biliary stenting compared to drainage alone [15]. There are other studies confirming this tendency [16–21].

To date no comparative data are available on patients, who received chemotherapy alone or a combination of PDT or TACE and chemotherapy.

The aim of this retrospective study was to explore and compare data of a selective patient group with bile duct tumours treated with various forms of therapy regarding their effect on morbidity and mortality. All patients were treated in the Gastroenterological Day Clinic of Medizinische Hochschule Hannover (MHH). 

## 2. Material and Methods

### 2.1. Data Collection

All patients undergoing treatment for a malignant bile duct tumour in the Gastroenterological Day Clinic of MHH between 1999 and 2005 were included in a retrospective analysis. The followup covered the period from diagnosis to death or last contact with the day clinic. Patient data were obtained from the MHH documentation system ALIDA and the H.I.T. data bank of the MHH Tumour Centre and entered into an individual own data bank (Microsoft Excel 2003, Germany).

The following data were collected: gender, date of birth, date of diagnosis and death, or date of last contact with the patient, height, body weight, BMI, localisation of the tumour, histology, histological/cytological confirmation, presence of various risk factors and symptoms, UICC/TNM classification, tumour markers CEA and CA 19-9, laboratory parameter (bilirubin, Quick, albumin, cholinesterase, GGT, ALT, and AST), previous operations and R-classification, and reason of death.

The patients were divided into two subgroups according to tumour localisation (ICD) and therapy distinguishing between patients with malignant tumours of the intrahepatic (C22.1) and extrahepatic bile ducts (C24.0), particularly Klatskin tumours of the hepatic duct bifurcation according to Bismuth.

### 2.2. Therapy

Generally, the patients received gemcitabine i.v. as chemotherapeutic drug. Alternatively, a combination of gemcitabine and cisplatin, gemcitabine, and oxaliplatin, 5-FU or 5-FU and oxaliplatin was applied.

Photodynamic therapy was performed in 3 sessions at 6 week intervals via ERC or PTCD according to the procedure established by Ortner/Berr et al. All patients received the photosensitizing agent Photofrin.

TACE was performed in the Radiological Department of MHH. After fasting a catheter was inserted into the patients' femoral artery and under radiological control an embolizing agent was injected into the blood vessel supplying the tumour. 

### 2.3. Statistics

The Kaplan-Meier survival curves were produced using PASW 18.0.2 (SPSS, *Somers/NY*, USA), and the prognosis in the subgroups compared using log rank test. The patients were divided into two subgroups (surgery versus palliation) and uni- and multivariate analysis of hazard ratios was performed using Cox regression. A *P*-value <  .05 showed significancy. Because of the small numbers of patients in each subgroup it was difficult to show a valid differentiated and stable multivariate analysis. The complete followup was surveyed, and in view of the known short life expectancy after diagnosis a 3-year survival rate was also determined and the remaining patients were censored.

## 3. Results

As Table 1 shows a total of 195 patients were included in the study, 84 females (43.1%) and 111 males (56.9%). At the time of diagnosis the median age was 58.47 years (females: 56.82 years, males: 59.7 years.); at the time of death the median age was 59.62 years (females 58.12 years, males 60.71 years). 

111 patients (56.9%) suffered from a tumour of the intrahepatic bile ducts (ICD C22.1) and 84 patients (43.1%) had an extrahepatic tumour (ICD C 24.0), for example, at the hepatic bifurcation (according to Bismuth).

Initially 3.7% (females 5.6%, males 2.2%) were at UICC stage IA, 14.6% (females: 19.4%, males: 10.9%) at stage IB, 19.5% (females: 19.4%, males: 19.6%) at stage IIA, 20.7% (females: 19.4%, males: 21.7%) at stage IIB, 11.0% (females: 13.9%, males: 8.7%) at stage III, and finally 30.5% (females: 22.2%, males: 37.0%) at stage IV. 

The average survival after diagnosis was 15.6 months (females: 17.43 months, males: 14.43 months). Three months after commencement of treatment, 165 (84.6%) of the 195 patients had survived (88.1% of females, 82.0% of males). After 6 months the surviving number of patients was reduced to 140 (71.8% females of 77.4%; males 67.6%), and after 1 year only half of all patients (50.8% females of 56.0; males 46.8%) surveyed since commencement of treatment had survived.

Around a quarter (26.5%) of the male patients and a third (33.8%) of the female patients suffered from a preexisting cholelithiasis. 11.8% of the males and 6.8% of the females suffered from primary sclerosing cholangitis and 5.8% of the males and 4.1% of the females suffered from ulcerative colitis. Liver cirrhosis was diagnosed in 15.5% of the males and in 8.0% of the females. 1.0% of the males and 4.1% of the females suffered from primary biliary cirrhosis. During the course of the treatment mild or moderate ascites was diagnosed in 17.5% of the males and 14.2% of the females. Thrombosis of the portal vein was seen in 6.2% of male patients and 12.7% of female patients.

In the majority of patients serum concentrations of bilirubin, cholinesterase, and coagulation parameters were within normal; the albumin concentration was reduced in one third (31.5%) of the females and a quarter (25.9%) of the males. The mean value determined for CEA was 21 *μ*g/L (standard deviation ±84 *μ*g/L) and CA 19-9 1521 U/L (standard deviation 3588 U/L) in males. For females the value determined for CEA was 8 *μ*g/L (standard deviation 27 *μ*g/L) and 13161 U/L (standard deviation 84518 U/L) for CA 19-9. Transaminases and GGT were elevated in all patients.

63 (34.1%) patients had previously undergone surgery, 15 (8.2%) patients received additional perioperative adjuvant chemotherapy. 

137 (80.6%) patients (60 females, 77 males) received chemotherapy in the Gastroenterological Day Clinic. 14 (7.7%) patients (6 females, 8 males) were treated with photodynamic therapy (PDT), and 18 (9.8%) patients (12 females. 6 males) were treated with transarterial chemoembolization. PDT was performed in 2 sessions (median, range 1–4 sessions).

However, at the end of the observation period, the result in the vast majority of patients (76.1%) was progression of the tumour disease. In 6% of the patients (approximately twice as many females as males) the tumour was no longer visible. Complete remission could be assumed in 3.3% and partial remission in 2.2% of the patients. In 3.3% a reduction of the tumour mass without defined remission could be assumed. In 4.9% no effect of the primary treatment was observed.

At the end of the observation period 80.6% of patients had died independent of gender.

93.5% (females 95.4%, males 92.2%) of all patients died by progression of the tumour's disease while only 1.9% (females: 0%, males: 3.3%) died by cardiovascular diseases (e.g., heart attack, stroke etc.). 4.5% (females 4.6%, males 4.4%) died by other diseases like sudden death of unknown reason, infection, liver failure, and so forth. 

If death was caused by tumour progression 42.9% (females: 40.0%, males: 44.4%) of all patients being evaluated by UICC—classification was at UICC stage IV ((IA 3.6% (females: 5.0%, males: 2.8%), IB 7,1% (females: 10.0%, males: 5.6%), IIA 23.2% (females: 25.0%, males: 22.2%), IIB 16.1% (females: 10.0%, males: 19.4%), III 7.1% (females: 10.0%, males: 5.6%)). The Cox regression analysis showed a higher hazard ratio for patients with UICC stage IIa and more. The risk was significantly higher after univariate analysis (*P* = .008) and insignificantly higher after multivariate analysis (*P* = .18).

The median survival time of patients, who had undergone surgery was 27.1 months, that is, the survival time was 17.1 months longer compared to those patients without primary surgical treatment (*P* < .01). Cox regression analysis additionally showed the hazard ratio was minimized significantly by this treatment (univariate *P* < .0001/multivariate *P* = .029). With adjuvant chemotherapy a median survival time of 33.4 months (*P* < .49) could be achieved, however, this was statistically insignificant (Table 2). The hazard ratio was significantly lower in univariate but not in multivariate analysis (*P* = .005/*P* = .18) (Figure 3).

A comparison of all patients (operated or palliative care) who were treated with chemotherapy in the Gastroenterological Day Clinic and patients without chemotherapy (Table 2) revealed that chemotherapy prolonged survival by a median of 9.2 months, which was statistically insignificant (*P* = .47). Univariate Cox regression showed a reduction of hazard ratio but without significancy (*P* = .47) while multivariate analysis showed a higher risk for patients undergoing chemotherapy which was also insignificant (*P* = .25) (Figure 3). As in the majority of cases patients with gastrointestinal tumours were treated with gemcitabine and the number of patients treated with an alternative substance was low, the Kaplan-Meier analysis distinguished only these two subgroups. First-line chemotherapy with gemcitabine revealed no advantage compared to other substances. In fact, there were statistically significant advantages for patients treated with alternative substances (*P* < .05). 

Concerning the interventional therapy groups it was noted that patients, who underwent photodynamic therapy survived 19.3 months compared to 15.5 months without photodynamic treatment. Again, there was no statistical significance (*P* = .49). Uni- and multivariate analysis showed a risk reduction which was not significant (*P* = .49/*P* = .52). In the group treated with transarterial chemoembolization survival was insignificantly prolonged by 7.5 months (*P* = .19) (Table 2) and the hazard ratio was also insignificantly lowered (*P* = .19/*P* = .25) (Figure 3). 

When looking at the various factors, which could have an influence on the average 3-year survival rate, marked formation of ascites (*P* < .001), previously present cholelithiasis (<.05), metastases (*P* < .001), reduced albumin concentration (*P* < .05), and reduced concentration of cholinesterase (<.05), elevated GGT (*P* < .01) and CEA (*P* < .05) were identified. The Cox regression analysis showed the hazard ratio was insignificantly higher for patients at an age of >60 years (*P* = .062/*P* = .62) but insignificantly lower for women (*P* = .2/*P* = .5)

**Figure 1:**
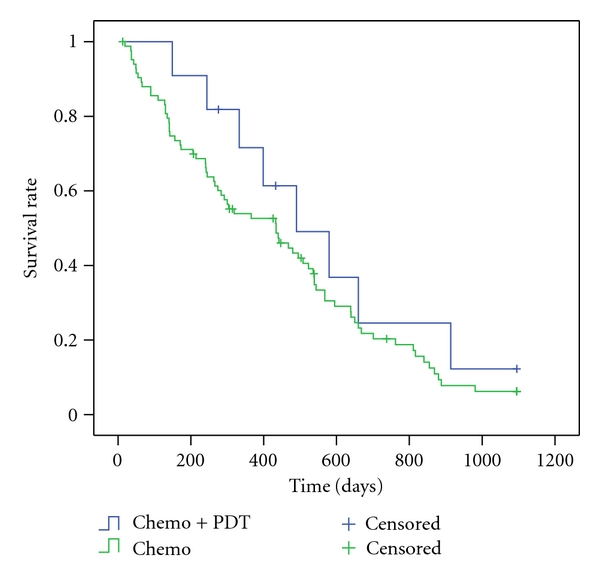
3-years-Kaplan-Meier estimate for chemo versus photodynamic therapy (*P* = .28) in patients with cholangiocarcinoma (palliation group).

**Figure 2:**
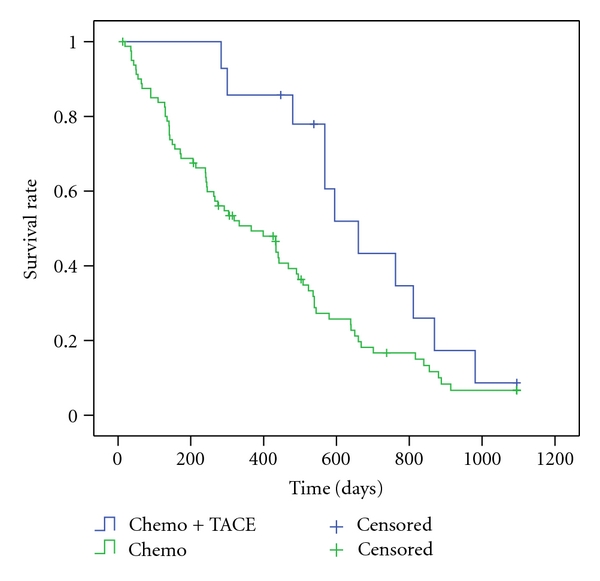
3-years-Kaplan-Meier estimate for chemo versus transarterial chemoembolisation (*P* = .04) in patients with cholangiocarcinoma (palliation group).

**Figure 3:**
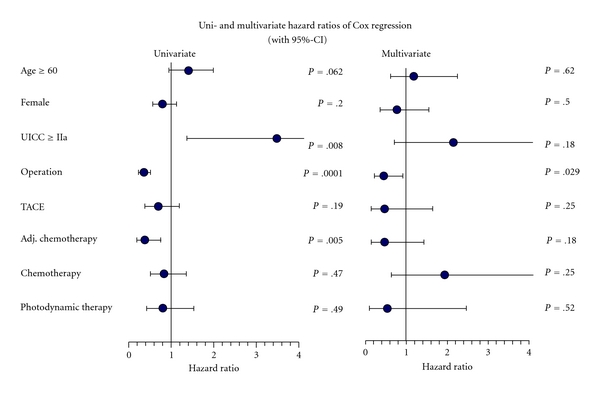
Uni- and multivariate analysis of hazard ratios by Cox regression method for all patients with cholangiocarcinoma.

As several studies dealing with the treatment of CCC patients with photodynamic therapy and stenting, but there are few data available on a combination of chemotherapy and photodynamic therapy or TACE, these patients were analysed separately in a “palliation only group” (*n* = 95). Patients under chemotherapy had a lower risk both in univariate and multivariate Cox regression (*P* < .001/*P* < .05) (Table 3). Median survival was 11.7 months longer than without chemotherapy (*P* < .05). Patients treated with a combination of chemo- and photodynamic therapy survived 1.8 months longer than those treated with chemotherapy alone (Table 3). However, there was no statistical significant difference (*P* = .28) (Figure 1) both in Kaplan-Meier-analysis and Cox regression (univariate/multivariate *P* = .23). The result for patients treated with a combination of TACE and chemotherapy was similar (Figure 2). Additional transarterial chemoembolization prolonged the survival time by 9.8 months, which has statistical significance (*P* < .05) in Kaplan-Meier-analysis (Table 3) and univariate Cox regression (*P* < .05) but not in multivariate analysis (*P* = .06). 

## 4. Discussion

To date no standard therapy has been established for patients with malignant tumours of the bile ducts. As this is a disease with a low but increasing incidence rate, the majority of patients are treated in clinical trials with protocols which vary considerably. In the majority of cases the disease is not diagnosed in the advanced stage. This complicates planning the therapy, which subsequently is palliative in most cases.

Our results partially correspond with those reported in numerous publications. The short survival time of 1 year and 3 months from diagnosis to death correlates with the survival times reported in known studies [22–24]. The high mortality rate with 50% within 1 year after diagnosis emphasises the great malignity of these tumours, and the currently mostly futile therapeutic efforts to achieve long-term remission or cure.

The association of significantly elevated or reduced values for cholinesterase, albumin GGT, and CEA with a shorter survival time can be explained by the severely impaired organ function in the advanced stage of the disease. Marked ascites and metastases are to be seen in the same context as uni- and multivariate analysis of the UICC stages additionally show. The survival was significantly reduced in patients, who suffered from cholelithiasis prior to the diagnosis of CCC. The predominant opinion is that chronic inflammatory stimulation of the cells caused by the permanent effect of bile acid is to be considered potentially malignant and influence the development of CCC [23, 25, 26]. 

Surgical treatment significantly improved the survival rate of patients treated at MHH. Kahn et al. [23], Yamamoto et al. [27], and Chen et al. [28] could already show similar results in three studies on surgical resection of CCC. Therefore, surgical resection remains to present the only sensible therapeutic measure with a curative approach at present. Adjuvant chemotherapy prolonged the survival time by approximately 6 months, which is contrary to various reports [22]. Takada et al. [29] could not see any advantages of adjuvant chemotherapy in his frequently cited work. The most likely reason for our differing result could be the small case number (*n* = 63), particularly as the data were statistically significant (*P* < .05) only in univariate Cox regression analysis but insignificant in Kaplan-Meier (*P* = .49) and multivariate analysis (*P* = .18). 

There were only a small number of patients in our observation group suffering from liver cirrhosis additionally (15.5% of males, 8.0% of females). Since all patients could be classified as Child-Pugh-Score A, and today liver transplantation seems to be an option for patients with cholangiocarcinoma only in experimental settings [30] we treated them like all different. 

On average the survival time of patients, who in view of the hopeless prognosis underwent primary chemotherapy with gemcitabine or alternative substances in a palliative approach, was prolonged by approximately 12 months. However, these observations correspond with studies that could show an improved survival with similar therapeutic regimens [31]. As the number of patients was small, we decided to divide the patient collective into two groups, one gemcitabine group and one group treated with alternative substances, for example, 5-FU, oxaliplatin or cisplatin. This revealed a significant advantage for the alternative group (*P* = .05). The available studies on the application of various chemotherapeutics appear to be very heterogenic. A whole range of substances are used, but the case numbers are generally small. The majority of studies are affected by the lack of randomisation. Eckel and Schmidt performed a meta-analysis of all studies on chemotherapy for CCC. This showed that a combination of gemcitabine and cisplatin or oxaliplatin achieved the best response rate and the most effective control of tumour growth [10]. In this context the survival advantage observed in our patients, who were treated with chemotherapy other than gemcitabine monotherapy, appears conclusive. 

In recent years interventional therapies such as photodynamic therapy or TACE were used successfully in a small selective patient group, mostly in combination with stenting of obstructed bile ducts [10, 32–35] (Table 4). Due to the significantly prolonged survival of the patients the frequently cited study by Ortner was prematurely terminated. The study by Dumoulin at least confirmed that photodynamic therapy combined with stenting considerably improved quality of life. 

The patients we treated with PDT or TACE also benefitted from this interventional strategy. Combination of systemic chemotherapy and interventional therapy prolonged the survival time by a few months. Even if there was no statistical significance this result indicates an advantage of combined conservative and interventional therapy. However, this should be verified in larger studies as the validity is limited by the small number of patients and the retrospective design of this study.

Finally, many factors like missing randomisation can bias the results of a retrospective study, that is, the effect of an applied therapy. Observation bias may occur because of misclassification or recall mistakes and frequently a number of patients are lost to followup. Criteria to select patients for different kinds of treatments vary during the years and in different hospitals. Due to the nature of rare diseases the possibility of observation is limited. 

## 5. Conclusion

Due to the late diagnosis, low incidence rate and great malignity tumours of the bile duct are a great challenge to physician. Due to the frequent lack of randomisation many studies have only limited validity and thus complicate the search for a gold standard in the treatment of these tumours. 

The results shown in this retrospective study merge with the number of new studies indicating a favourable influence on survival of combined conservative and interventional procedures. However, it is difficult to obtain reliable data due to a small number of cases, a heterogenic patient group, and partially inadequate documentation. Future studies with larger patient groups for the therapeutic options presented are desirable, to achieve advances in the therapy of these highly malignant tumours.

## Figures and Tables

**Figure 4:**
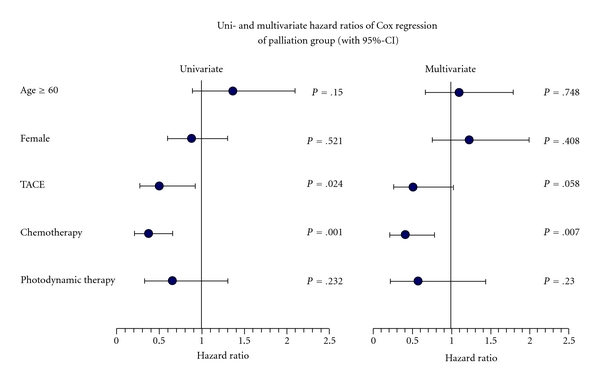
Uni- and multivariate analysis of hazard ratios by Cox regression method for patients with cholangiocarcinoma (palliation group).

**Table 1:** Age, localisation, therapies, and results of all patients with diagnosis of cholangiocarcinoma.

Variables	Total	(*n*)	Women	(*n*)	Men	(*n*)
		(*n* = 195)		(*n* = 84)		(*n* = 111)
*Median age at point of diagnosis*	58.47 (±12.28)		56.83 (±10.74)		59.70 (±13.24)	
*Median age at point of death*	59.62 (±11.97)		58.12 (±11.03)		60.71 (±12.57)	
*Localisation*						
C22.1	111 (56.9%)		48 (57.1%)		63 (56.8%)	
C24.0	84 (43.1%)		36 (42.9%)		48 (43.2%)	
*Therapy*						
OP	63 (34.1%)	(*n* = 185)	28 (35.4%)	(*n* = 79)	35 (33.0%)	(*n* = 106)
adjuvant chemotherapy	15 (8.2%)	(*n* = 183)	7 (9.0%)	(*n* = 78)	8 (7.6%)	(*n* = 105)
photodynamic therapy	14 (7.7%)	(*n* = 183)	6 (7.6%)	(*n* = 79)	8 (7.7%)	(*n* = 104)
TACE	18 (9.8%)	(*n* = 183)	12 (15.2%)	(*n* = 79)	6 (5.8%)	(*n* = 104)
chemotherapy	137 (80.6%)	(*n* = 170)	60 (83.3%)	(*n* = 72)	77 (78.6%)	(*n* = 98)
*Results of therapy*		(*n* = 184)		(*n* = 81)		(*n* = 103)
no tumor	11 (6.0%)		7 (8.6%)		4 (3.9%)	
complete remission	6 (3.3%)		4 (4.9%)		2 (1.9%)	
partial remission	4 (2.2%)		1 (1.2%)		3 (2.9%)	
Reduction of tumor mass without def. Rem.	6 (3.3%)		1 (1.2%)		5 (4.9%)	
unchanged	8 (4.3%)		2 (2.5%)		6 (5.8%)	
progress	140 (76.1%)		62 (76.5%)		78 (75.7%)	
no result of primary therapy	9 (4.9%)		4 (4.9%)		5 (4.9%)	
*Survival after*		(*n* = 195)		(*n* = 84)		(*n* = 111)
3 months	165 (84.6%)		74 (88.1%)		91 (82.0%)	
6 months	140 (71.8%)		65 (77.4%)		75 (67.6%)	
12 months	99 (50.8%)		47 (56.0%)		52 (46.8%)	
*Event of death*	154 (80.6%)	(*n* = 191)	65 (80.2%)	(*n* = 81)	89 (80.9%)	(*n* = 110)

**Table 2:** Median 3-year survival (IQR) under conservative versus interventional therapies including all patients with cholangiocarcinoma.

Therapy	Total (*n*)	Median survival (months)	Significance (*P*)
OP	61	27.1 (14.9)	
No OP	121	10.0 (15.1)	<.001
Adjuvant chemotherapy	14	33.4 (27.1)	
No adj. chemo	47	26.6 (14.9)	.49
Chemotherapy	136	17.4 (20.4)	
No chemotherapy	33	8.2 (3)	.466
PDT	14	19.3 (19.4)	
No PDT	166	15.5 (27.3)	.488
TACE	18	22.0 (16.7)	
No TACE	162	14.5 (22.8)	.190

**Table 3:** Median 3-year-survival (IQR) of patients with cholangiocarcinoma undergoing stand-alone chemotherapy versus combination of chemotherapy and interventional therapies (palliation group).

Therapy	Total (*n*)	Median survival (months)	significance (*P*)
Chemo + PDT	11	16.3 (10.9)	
Chemo	84	14.5 (16.9)	.283
Chemo + TACE	14	22.0 (10)	
Chemo	81	12.2(16.6)	.039

**Table 4:** Series published on PDT.

Author	Year	Patients (*n*)	Median survival (months)
Ortner et al.	1998	9	14.4 (3.0–18.9)
Berr et al.	2000	23	11.1 (0.8–50.7)
Rumalla et al.	2001	6	>9
Dumoulin et al.	2003	24	9.9 (2–39)
Ortner et al.	2003	20	16.3 (9.1–23.3)
Zoepf et al.	2005	16	21
Shim et al.	2005	24	18.6 (2–27)

Total		188	9.9–21
